# BoaBZR1.1 mediates brassinosteroid-induced carotenoid biosynthesis in Chinese kale

**DOI:** 10.1093/hr/uhae104

**Published:** 2024-04-09

**Authors:** Chenlu Zhang, Qiannan Liang, Yilin Wang, Sha Liang, Zhi Huang, Huanxiu Li, Victor Hugo Escalona, Xingwei Yao, Wenjuan Cheng, Zhifeng Chen, Fen Zhang, Qiaomei Wang, Yi Tang, Bo Sun

**Affiliations:** College of Horticulture, Sichuan Agricultural University, Chengdu 611130, China; College of Horticulture, Sichuan Agricultural University, Chengdu 611130, China; College of Horticulture, Sichuan Agricultural University, Chengdu 611130, China; College of Horticulture, Sichuan Agricultural University, Chengdu 611130, China; College of Horticulture, Sichuan Agricultural University, Chengdu 611130, China; College of Horticulture, Sichuan Agricultural University, Chengdu 611130, China; Faculty of Agricultural Sciences, University of Chile, Santiago 8820000, Metropolitan Region, Chile; State Key Laboratory of Vegetable Biobreeding, Tianjin Academy of Agricultural Sciences, Tianjin, 300192, China; Tianjin Academy of Agricultural Sciences, Tianjin, 300192, China; State Key Laboratory of Vegetable Biobreeding, Tianjin Academy of Agricultural Sciences, Tianjin, 300192, China; Tianjin Academy of Agricultural Sciences, Tianjin, 300192, China; College of Biology and Agriculture Technology, Zunyi Normal University, Zunyi 563000, China; College of Horticulture, Sichuan Agricultural University, Chengdu 611130, China; Department of Horticulture, Zhejiang University, Hangzhou 310058, China; College of Horticulture, Sichuan Agricultural University, Chengdu 611130, China; College of Horticulture, Sichuan Agricultural University, Chengdu 611130, China

## Abstract

Brassinazole resistant 1 (BZR1), a brassinosteroid (BR) signaling component, plays a pivotal role in regulating numerous specific developmental processes. Our study demonstrated that exogenous treatment with 2,4-epibrassinolide (EBR) significantly enhanced the accumulation of carotenoids and chlorophylls in Chinese kale (*Brassica oleracea var. alboglabra*). The underlying mechanism was deciphered through yeast one-hybrid (Y1H) and dual-luciferase (LUC) assays, whereby BoaBZR1.1 directly interacts with the promoters of *BoaCRTISO* and *BoaPSY2*, activating their expression. This effect was further validated through overexpression of *BoaBZR1.1* in Chinese kale calli and plants, both of which exhibited increased carotenoid accumulation. Additionally, qPCR analysis unveiled upregulation of carotenoid and chlorophyll biosynthetic genes in the T1 generation of *BoaBZR1.1*-overexpressing plants. These findings underscored the significance of BoaBZR1.1-mediated BR signaling in regulating carotenoid accumulation in Chinese kale and suggested the potential for enhancing the nutritional quality of Chinese kale through genetic engineering of BoaBZR1.1.

## Introduction

As a Cruciferae vegetable, Chinese kale (*Brassica oleracea var. alboglabra*) is native to South China, which is the global center of its diversity [1, 2]. Primarily consumed for its leaves and bolting stems, Chinese kale is notable for its abundance of health-promoting compounds, including ascorbic acid, glucosinolates, and carotenoids [3]. Our study has investigated carotenoid variations in different Chinese kale varieties and organs, and we observed a significant richness of carotenoids in Chinese kale leaves, confirming the potential of Chinese kale as an excellent source of carotenoids [4].

Carotenoids, typically comprised of eight isoprene units, belong to the terpenoid family and serve as prevalent natural pigments in plants [5–7]. In plants, carotenoids play vital roles in light capture, photosynthetic protection, and contribute to the vibrant red, orange, and yellow hues, along with aroma and flavor [8–10]. For human health, carotenoids are essential nutrients and have witnessed growing utilization in dietary supplements and pharmaceuticals in recent years. β-carotene, serving as a precursor to vitamin A, is widely employed in preventing and treating night blindness [6, 11]. Additionally, carotenoids enhance the body’s antioxidant capacity, playing a crucial role in anti-aging, cancer prevention, and reducing the risk of chronic diseases like cardiovascular and cerebrovascular diseases [12, 13]. Because the human body lacks carotenoid biosynthetic enzymes, dietary supplementation of carotenoids is imperative for human health [14]. Consequently, enhancing carotenoid content in crops such as grains, fruits, and vegetables through molecular breeding techniques has emerged as a prominent research focus.

Presently, the carotenoid biosynthesis pathway in model plants such as *Arabidopsis* and tomato has been extensively elucidated [15, 16]. CRTISO, a pivotal enzyme preceding the bifurcation point in the carotenoid biosynthesis pathway, catalyzes the conversion of lycopene precursors into lycopene [17]. Prior research indicated that the loss of *CRTISO* function in crops like tomato and rice resulted in crop yellowing [18, 19]. Mutations in *CRTISO* in *Brassica napus* led to the change of petal color from yellow to white and leave color from green to yellow-green [20]. Similarly, in our previous study, a decrease in both carotenoid and chlorophyll contents was noted in Chinese kale leaves following the editing of the *BoaCRTISO* gene using CRISPR/Cas9 [3].

Brassinosteroids (BRs) are essential steroid hormones governing plant processes such as growth, development, cell elongation, and responses to both biotic and abiotic stress [21]. The BR signaling pathway has undergone extensive analysis. This process entails the activation of the BR signal receptor BRI1, the inhibition of the negative regulatory factor BIN2, and the subsequent induction of positive regulatory factors such as BZR1 and BES1. Ultimately, these events regulate specific physiological processes [22]. Research has demonstrated that BR can enhance carotenoid accumulation in plants. Sang *et al.* reported an elevation in lycopene content in tomato fruit following treatment with exogenous 28-homobrassinolide (HBR) [23]. In our earlier study, we observed a substantial increase in total carotenoids content in Chinese kale sprouts following epi-brassinolide (eBL) treatment [24].

BZR1 serves as a recognized positive regulator of the BR signal, enhancing carotenoid biosynthesis in plants [25]. In tomato fruits, overexpression of *SlBZR1* activated the expression of *SlPSY1*, promoted the accumulation of individual and total carotenoids and accelerated the ripening of tomato fruits [26]. Additionally, the APETALA2a/DWARF/BZR1 complex integrated ethylene and BR signals to enhance lycopene accumulation and accelerate fruit ripening [23]. However, research on the role of BZR1 in promoting carotenoid biosynthesis predominantly concentrates on fruit vegetables like tomatoes, with limited reporting on how BZR1 regulates carotenoid biosynthesis in green leafy vegetables.

In this study, we observed that exogenous BR treatment substantially increased the accumulation of both carotenoids and chlorophylls in Chinese kale. This contrasted with the findings in tomato fruits, where exogenous BR treatment promoted carotenoid accumulation but accelerated chlorophyll degradation [27]. Furthermore, we elucidated how the transcription factor BoaBZR1.1 mediates BR-regulated carotenoid biosynthesis through activating the expression of *BoaCRTISO*. Our findings will contribute to understanding the molecular mechanism of positive regulation of carotenoid biosynthesis by BR, providing a theoretical foundation for breeding high-carotenoid Chinese kale cultivars. This study has the potential to enhance the economic prospects of Chinese kale and advance the *Brassica* vegetable industry.

## Results

### BR promoted the accumulation of carotenoids and chlorophylls in Chinese kale

**Figure 1:**
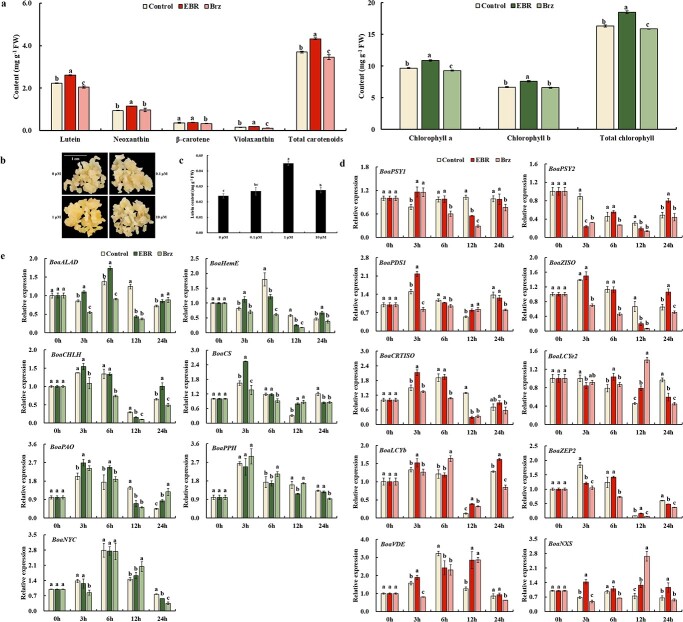
Effects of exogenous BR or Brz treatments on carotenoid biosynthesis in Chinese kale. **a** Changes of carotenoid and chlorophyll contents in Chinese kale plants after exogenous BR or Brz treatments. **b** Color of Chinese kale calli after exogenous EBR treatment. **c** Exogenous EBR treatment increased lutein content in calli of Chinese kale. **d** Exogenous EBR treatment promoted the expression of carotenoid biosynthetic genes in Chinese kale plants. **e** Exogenous EBR treatment promoted the expression of chlorophyll biosynthetic genes in Chinese kale plants. Bars are means ± SD of three biological replicates. The same letter in the same histogram indicates that there is no significant difference between the values tested by least significant difference (LSD) (*p* < 0.05).

The carotenoid and chlorophyll contents in Chinese kale were assessed after treatment with water, EBR, and Brz, respectively. Lutein and neoxanthin were the predominant carotenoids, comprising over 50% and 20% of the total carotenoids. EBR treatment markedly elevated total and individual carotenoids except for β-carotene (Fig. 1a). Similarly, the carotenoids content in Chinese kale calli grown on media supplemented with 0.1 μM, 1 μM, and 10 μM EBR was measured respectively, with the calli grown on media without EBR as control. The results showed that the calli grown on media containing 1 μM EBR exhibited a more intense yellow coloration (Fig. 1b). The further HPLC analysis revealed that only lutein was detected in Chinese kale calli, and treatment with 1 μM EBR significantly increased the lutein content in the calli of Chinese kale (Fig. 1c), which was consistent with the results that 1 μM is the most effective concentration for BL to inhibit anthocyanin and proanthocyanidin in apple calli [28, 29]. In contrast to EBR treatment, Brz treatment led to a significant reduction in total and individual carotenoid content except for neoxanthin. In addition, for chlorophyll, the application of EBR and Brz promoted and inhibited the accumulation of total chlorophylls and individual chlorophyll, respectively (Fig. 1a). These findings indicated that BR stimulated the accumulation of carotenoids and chlorophylls in Chinese kale.

Furthermore, we examined the impact of various treatments on the expression of carotenoid and chlorophyll biosynthetic genes in Chinese kale. Following EBR treatment, the expression of carotenoid biosynthetic genes exhibited an increasing trend within the first 3 to 6 hours, with the exception of *BoaPSY2* and *BoaLCYe2*, which did not display a discernible pattern of change. Notably, most carotenoid biosynthetic genes (*BoaPSY1*, *BoaPDS1*, *BoaCRTISO*, *BoaLCYb*, *BoaVDE*, and *BoaNXS*) exhibited a response to EBR treatment at 3 h, with *BoaPSY1*, *BoaPDS1*, and *BoaCRTISO* showing a particularly pronounced response, with fold changes of 1.49, 1.45, and 1.41 compared to the control, respectively. Treatment with Brz resulted in a substantial inhibition of most carotenoid biosynthetic genes. While *BoaPSY1* and *BoaCRTISO* did not exhibit a response to Brz until 6 hours, the inhibitory effects of Brz treatment on both genes persisted for 24 hours (Fig. 1d). These findings indicated that *BoaPSY1*, *BoaPDS1*, and *BoaCRTISO* display significant responses to both EBR and Brz treatments.

Regarding chlorophyll biosynthetic genes, their expression levels initially increased and subsequently decreased within the 0 to 12-hour period following EBR treatment. Specifically, *BoaCHLH* and *BoaCS* reached their peak expression at 3 hours, while *BoaALAD* and *BoaHemH* exhibited their highest expression levels at 6 hours. In contrast to EBR treatment, Brz treatment suppressed the expression of chlorophyll biosynthetic gene within 3–6 hours. For the chlorophyll degrading genes, only *BoaPAO* expression was promoted by EBR treatment, while *BoaPPH* and *BoaNYC* showed no significant response. Furthermore, Brz treatment did not exhibit a noticeable inhibitory effect on the expression of all three genes (Fig. 1e).

### BoaBZR1 was identified by yeast one-hybrid library screening

Due to the vital role of CRTISO in carotenoid biosynthesis and the significant enhancement of *BoaCRTISO* expression in response to EBR treatment, we employed *BoaCRTISO* promoter as a bait for yeast one-hybrid library screening. The *BoaCRTISO* promoter was divided into three segments, with segment 2 (pro-2) displaying self-activation (Fig. ure S1, see online supplementary material). Consequently, segments 1 (pro-1) and 3 (pro-3) were utilized in the yeast one-hybrid library screening, resulting in the identification of seven interacting proteins. Among these, BZR1 was selected for further in-depth exploration.

### Isolation and characterization of the BoaBZR1s

Three cabbage *BolBZR1* copies, *BolC02g031400*, *BolC06g046370*, and *BolC06g03106*, were retrieved from the *Brassica* Database (BRAD). Using these three sequences as a reference, the coding sequences of *BoaBZR1.1*, *BoaBZR1.2*, and *BoaBZR1.3* from Chinese kale cultivar ‘Sijicutiao’ were cloned, with lengths of 699 bp, 924 bp, and 1002 bp, respectively. A comparison of the amino acid sequences of BoaBZR1s revealed that these three BoaBZR1s share a sequence identity of 72.25%. In addition, all three BoaBZR1s sequences featured PEST and EAR domains, with BoaBZR1.2 and BoaBZR1.3 also containing BES1_N superfamily domains (Fig. ure S2a, see online supplementary material). Phylogenetic analysis revealed that BoaBZR1s formed a cluster with BZR1s from other *Brassica* species, with the highest similarity observed between BoaBZR1s and BolBZR1s from cabbage, underscoring the high conservation of BZR1s within the *Brassica* genus (Fig. ure S2b, see online supplementary material).

**Figure 2:**
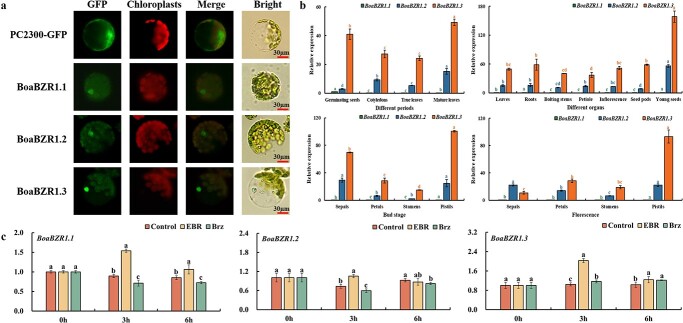
Subcellular localization (**a**), temporal and spatial expression of *BoaBZR1s* (**b**) and their responses to exogenous BR/Bzr treatments (**c**). Bars = 30 μm. Bars are means ± SD of three biological replicates. The same colors of letter represent the same genes, and the same letter in the same histogram indicates that there is no significant difference between the values tested by least significant difference (LSD) (*P* < 0.05).

In order to analyse the subcellular localization of BoaBZR1s, we first predicted the nuclear localization signals (NLSs) of BoaBZR1s online (http://www.moseslab.csb.utoronto.ca/NLStradamus/). No NLS was found in BoaBZR1.1, while the sequence between amino acids 17 and 41 (^17^RRKPSWRERENNRRRERRRRAIAAK^41^) of BoaBZR1.2, and the sequence between amino acids 20 and 44 (^20^RRKPSWRERENNRRRERRRRAVAAK^44^) of BoaBZR1.3 were found, which were highly similar to the NLS of AtBZR1 (^20^AARRKPSWRERENNRRRERRRRAV^43^) [30]. Then, a transient overexpression system was utilized in Chinese kale protoplasts to investigate the cellular location of BoaBZR1s. When BoaBZR1s proteins were expressed in GFP fusions under the regulation of the *Cauliflower mosaic virus* (CaMV) 35S promoter, the resulting fluorescent signals clearly demonstrated exclusively localization within the nucleus, thus confirming the nuclear presence of BoaBZR1s (Fig. 2a).

### Different expression patterns of *BoaBZR1s* in Chinese kale

The expression patterns of *BoaBZR1.1*, *BoaBZR1.2*, and *BoaBZR1.3* were different in different periods and organs of Chinese kale. Notably, *BoaBZR1.1* exhibited the lowest transcription level, while *BoaBZR1.3* displayed the highest (except in sepals at the flowering stage). During the growth and development of Chinese kale, the trends of the expression alteration of *BoaBZR1.1*, *BoaBZR1.2*, and *BoaBZR1.3* were different. The expression level of *BoaBZR1.1* in germinating seeds exhibited the highest magnitude, significantly surpassing that in cotyledons, true leaves, and mature leaves; whereas the expression levels of *BoaBZR1.2* and *BoaBZR1.3* reached their peak in mature leaves. The expression levels of *BoaBZR1s* in seven different organs were compared. It was found that the expression levels of three *BoaBZR1*s genes in young seeds were significantly higher than those in other organs. In flower organs, both of the highest expression levels of *BoaBZR1.1* and *BoaBZR1.3* were found in stamens, while *BoaBZR1.2* had the highest expression in sepals (Fig. 2b).

### Exogenous BR treatment induced the expression of *BoaBZR1s* in Chinese kale.

To gain a deeper understanding of BoaBZR1s’ role in BR signaling, we investigated whether BR influences the transcription levels of *BoaBZR1s*. The qPCR analysis showed that EBR treatment triggered an upregulated expression of *BoaBZR1s*, with all three isoforms peaking at 3 hours within the 0 to 6-hour timeframe. In contrast to the EBR treatment, the expression levels *BoaBZR1.1* and *BoaBZR1.2* were downregulated following Brz treatment (Fig. 2c). These findings confirm that exogenous BR treatment induces the expression of *BoaBZR1s* in Chinese kale.

### BoaBZR1.1 directly activated the expression of *BoaCRTISO* and *BoaPSY2*

**Figure 3:**
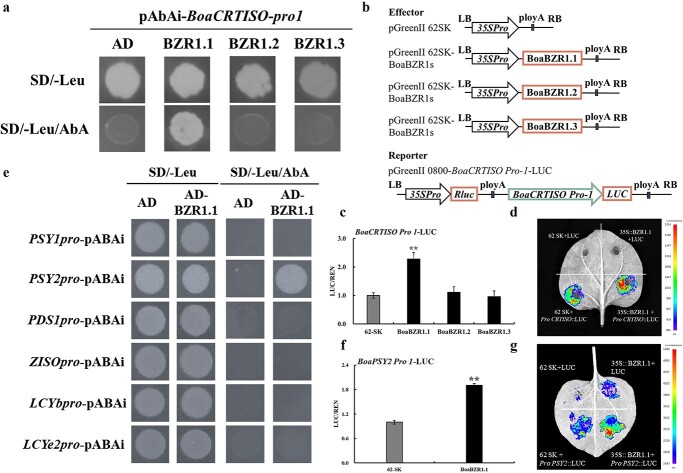
BoaBZR1.1 directly interacted with the promoter of *BoaCRTISO* and *BoaPSY2* to activate their expression. **a** Y1H assays verified the interaction between BoaBZR1.1 and the *BoaCRTISO* promoter. **b** Construction of dual-luciferase reporter system vector for BoaBZR1s and *BoaCRTISO* promoter. **c** Ratio of firefly luciferase (LUC) and renilla luciferase (REN) of Pro 35 s: 62-SK+ *Pro CRTISO*::LUC was set as 1. Bars are means ± SD of three biological replicates. Asterisks indicate significant differences between negative control and BoaBZR1.1-SK + *Pro CRTISO*::LUC (**P ≤ 0.01). **d** Dual-luciferase reporter assay results reflecting the transcriptional activation of *BoaCRTISO* by BoaBZR1.1. **e** Y1H assay showed that BoaBZR1.1 can only directly bind to the promoter of *BoaPSY2*. **f** Ratio of firefly luciferase (LUC) and renilla luciferase (REN) of Pro 35 s: 62-SK+ *Pro PSY2*::LUC was set as 1. Bars are means ± SD of three biological replicates. Asterisks indicate significant differences between negative control and BoaBZR1.1-SK + *Pro CRTISO*::LUC (**P ≤ 0.01). **g** Dual-luciferase reporter assay results reflecting the transcriptional activation of *BoaPSY2* by BoaBZR1.1.

To investigate whether BoaBZR1s directly regulate *BoaCRTISO* transcription, a yeast one-hybrid (Y1H) assay was initially conducted to evaluate the interaction between BoaBZR1.1, BoaBZR1.2, BoaBZR1.3, and the *BoaCRTISO* promoter. As shown in Fig. 3a, yeast cells co-transformed with *BoaCRTISO*-*pro1* and AD-BoaBZR1.1 were the only ones able to grow on SD/−Leu/AbA medium. This indicated exclusive binding of BoaBZR1.1 to the *BoaCRTISO* promoter, while BoaBZR1.2 and BoaBZR1.3 showed no direct impact on *BoaCRTISO*. Subsequently, a dual-luciferase reporter assay was employed to delve deeper into the regulatory relationship between BoaBZR1s and *BoaCRTISO* (Fig. 3b). As shown in Fig. 3c and d, co-expression of BoaBZR1.1 and *BoaCRTISO Pro 1*-LUC resulted in a notable increase in luminescence intensity. On the contrary, BoaBZR1.2 or BoaBZR1.3 did not elicit the expression of *BoaCRTISO*  *Pro 1*-LUC.

In order to further understand the regulation of BoaBZR1.1 on carotenoid biosynthesis of Chinese kale, we conducted Y1H assay to verify whether BoaBZR1.1 can directly regulate other carotenoid biosynthesis genes besides *BoaCRTISO*. Fig. 3e demonstrates that only yeast cells co-transformed with *BoaPSY2*-*pro* and AD-BoaBZR1.1 were able to grow on SD/−Leu/AbA medium, indicating that among the selected carotenoid biosynthetic genes, only *BoaPSY2* was directly regulated by BoaBZR1.1. Furthermore, a dual-luciferase reporter assay revealed that BoaBZR1.1 activated the expression of *BoaPSY2* (Fig. 3f and g). Collectively, these results suggested that BoaBZR1.1 positively regulates carotenoid biosynthesis by directly targeting the promoters of both *BoaCRTISO* and *BoaPSY2* in Chinese kale.

### Overexpressing *BoaBZR1.1* increased lutein content in Chinese kale calli

**Figure 4:**
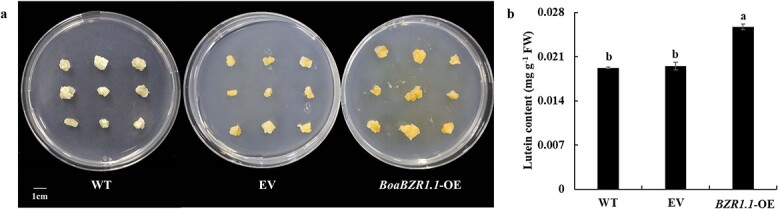
BoaBZR1.1 increased the lutein content in Chinese kale calli. **a** Phenotypes of transgenic Chinese kale calli. **b** Content of lutein in transgenic Chinese kale calli. WT: Chinese kale calli without transforming *Agrobacterium tumefaciens*; EV: Chinese kale calli that transferred by empty vector; *BoaBZR1.1*-OE: Chinese kale calli that transferred by pCAMBIA1301-*BoaBZR1.1*. Bars are means ± SD of three biological replicates. The same letter in the same histogram indicates that there is no significant difference between the values tested by least significant difference (LSD) (*P* < 0.05).

In view of the fact that the regulation mechanism of *PSY* on carotenoid biosynthesis has been intensively studied [31, 32], we conducted a transient overexpression in Chinese kale calli and observed that the calli overexpressing *BoaBZR1.1* exhibited a deeper yellow hue compared to those untransformed and transformed with an empty vector (Fig. 4a). Subsequent HPLC analysis of carotenoid components and content revealed exclusive lutein accumulation. Notably, the overexpression of *BoaBZR1.1* significantly elevated the lutein content to 0.0257 mg g^−1^ FW, representing 1.34-fold and 1.31-fold of the calli untransformed (0.0192 mg g^−1^ FW) and transformed with the empty vector (0.0195 mg g^−1^ FW), respectively (Fig. 4b).

### *BoaBZR1.1* promoted the biosynthesis of carotenoids and chlorophylls in Chinese kale

Based on *Agrobacterium tumefaciens*-mediated genetic transformation of Chinese kale, three T0 generation transgenic lines of *BoaBZR1.1*-OE, *BoaBZR1.1*–9, *BoaBZR1.1*–17, and *BoaBZR1.1*–19, were obtained (Fig. ure S3a and b, see online supplementary material). Due to variations in the growth periods of T0 generation plants, the seeds of T0 generation were sown to obtain T1 generation. T1 generation plants were confirmed by specific primers targeting hygromycin (Hyg) and β-glucuronic acid enzyme gene (GUS) and exhibited the higher expression level of *BoaBZR1.1* compared with WT. The results proved that the overexpression of *BoaBZR1.1* was stably inherited (Fig. ure S3c, see online supplementary material).

**Figure 5:**
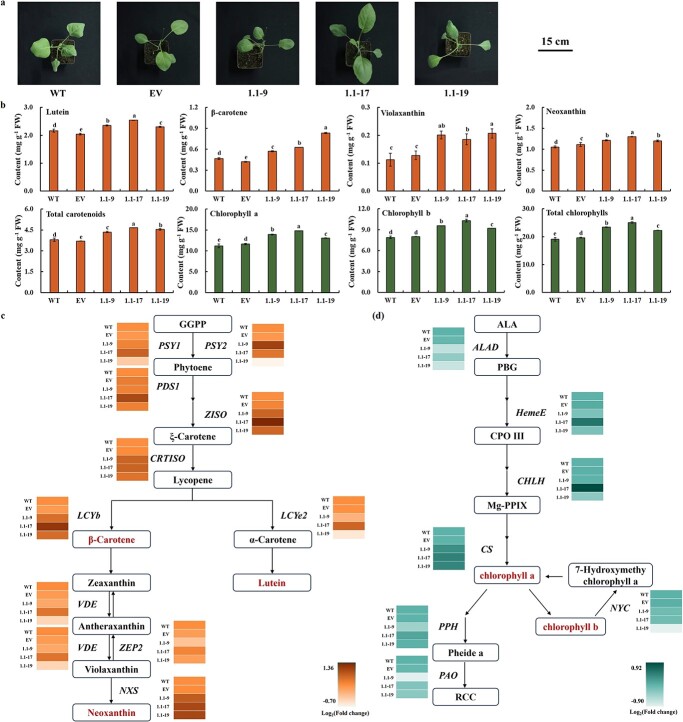
*BoaBZR1.1* promoted the biosynthesis of carotenoids and chlorophylls in Chinese kale. **a** Phenotypes of T1 generation of *BoaBZR1.1*-OE Chinese kale. **b** Contents of carotenoids and chlorophylls in T1 generation of *BoaBZR1.1*-OE Chinese kale. Bars are means ± SD of three biological replicates. The same letter in the same histogram indicates that there is no significant difference between the values tested by least significant difference (LSD) (*P* < 0.05). **c** Expression of carotenoid biosynthetic genes in T1 generation of *BoaBZR1.1*-OE Chinese kale. **d** Expression of chlorophyll biosynthetic and degrading genes in T1 generation of *BoaBZR1.1*-OE Chinese kale.

We observed and compared the difference in leaf color between WT and T1 generation of *BoaBZR1.1*-OE plants. All of the values of a*, b*, and L* of three *BoaBZR1.1*-OE plants were lower than those in WT, indicating an increase in the green hue of *BoaBZR1.1*-OE plants, accompanied by a decrease in the yellow hue and brightness (Fig. 5a; Fig. ure S3d, see online supplementary material). Then, the pigment content was measured, and T1 generation of *BoaBZR1.1*-OE plants showed elevated levels of both total and individual carotenoids and chlorophylls compared to the WT. Among them, *BoaBZR1.1*–17 exhibited the highest content of lutein, neoxanthin, and total carotenoids, with values 1.23, 1.17, and 1.24 times that of WT, respectively. The content of β-carotene and violaxanthin was the highest in *BoaBZR1.1*–19, which was 1.79 and 1.84 times that of WT. In term of chlorophyll, the T1 generation of *BoaBZR1.1*–17 exhibited the highest levels of chlorophyll a, chlorophyll b, and total chlorophylls, with values 1.32, 1.32, and 1.31 times that of WT, respectively. These results showed that overexpression of *BoaBZR1.1* significantly increased the contents of carotenoids and chlorophylls in Chinese kale (Fig. 5b).

The expression levels of pigment-related genes were also evaluated in the T1 generation of *BoaBZR1.1*-OE plants. As shown in Fig. 5c, mirroring the carotenoid content, the most elevated expression levels of all carotenoid biosynthesis-related genes (except *BoaPSY2*) were identified in *BoaBZR1.1*–17. Additionally, the expression levels of *BoaPDS1*, *BoaZISO*, *BoaCRTISO*, *BoaLCYb*, and *BoaNXS* were significantly higher in all three T1 generation strains than in WT. For chlorophyll biosynthetic genes, the expression of *BoaCS* significantly increased in all three *BoaBZR1.1*-OE strains, and *BoaCHLA* and *BoaHemE* exhibited significantly higher expression in *BoaBZR1.1*–17 compared to WT. Furthermore, the expression levels of chlorophyll degradation genes *BoaPao* and *BoaNYC* were down-regulated in *BoaBZR1.1*-OE plants (Fig. 5d). Collectively, these results suggested that the overexpression of *BoaBZR1.1* resulted in concurrent upregulation of carotenoid and chlorophyll biosynthetic genes, along with the downregulation of chlorophyll degradation genes, ultimately leading to the simultaneous accumulation of carotenoids and chlorophylls in Chinese kale plants overexpressing *BoaBZR1.1*.

## Discussion

The regulation of BR on carotenoid and chlorophyll biosynthesis may be different in different species and organs. In tomato fruit, exogenous BR treatment not only enhanced lycopene synthesis but also accelerated chlorophyll degradation, and finally accelerated the ripening and color change of fruits, making the ripe tomato fruits appear deep red [27]. However, in tomato seedlings, exogenous BR treatment increased the chlorophyll content, enhanced their photosynthesis and finally promoted their growth and development [33]. In *Arabidopsis* seedlings, treatment with exogenous brassinolide (BL) inhibited the accumulation of carotenoids and chlorophylls [34]. However, in this study, we found that BR could upregulate the expression of genes involved in carotenoid and chlorophyll biosynthesis, inhibit the transcription of chlorophyll-degrading genes, stimulate carotenoid and chlorophyll accumulation in Chinese kale simultaneously. These results in agreement with the findings in other *Brassica* plants that BR promoted carotenoid biosynthesis in Chinese kale sprouts and *Brassica juncea* leaves [24, 35], and increased chlorophyll content in wucai [36]. According to these results, we suggested that BR has a different regulation effect on the accumulation of carotenoids and chlorophylls in the process of vegetative growth and reproductive growth, and this effect is also different in different species.

Exogenous BR has a limited time to promote carotenoid biosynthesis in plants. In this study, compared with the control group, the expression of carotenoid biosynthesis genes was up-regulated at 3 h and 6 h after the EBR treatment to the Chinese kale, but decreased at 12 h and slightly increased at 24 h, which was similar to the result in the study of apple and *Arabidopsis thaliana* that the general pattern of expression for genes in BR-treated seedlings was to first increase and then decrease [37, 38]. In this way, exogenous BR only has a regulatory effect on plant gene expression in a period of time after treatment, and this effect gradually weakens or even disappears as the time of exogenous treatment went by. In addition, for different species and different ages of seedling, exogenous treatments had different durations of action. We suggested that the promoting effect of EBR on carotenoid biosynthesis genes in Chinese kale was mainly concentrated at 3–6 h, and that the enhanced effect of carotenoid biosynthesis may initiate a negative feedback regulatory mechanism in Chinese kale itself, which may be one of the reasons for the decreased expression of carotenoid biosynthetic genes at 12 h. Moreover, the circadian rhythm and photoperiod of photosynthesis may also affect the expression of carotenoid biosynthetic genes, resulting in the gene expression returning to its own level after the effect of EBR basically disappeared at 24 h.

The regulatory mechanism of BZR1 on carotenoid biosynthesis was also different in Chinese kale leaves and tomato fruits. In this study, BoaBZR1.1 directly interacted with the promoter of *BoaCRTISO* and *BoaPSY2*, activating their expression, and subsequently enhanced the biosynthesis of carotenoids, while other key genes in carotenoid biosynthesis, such as *BoaPDS*, *BoaZDS*, *BoaPSY1*, and *BoaPSY3*, were not directly regulated by BoaBZR1.1. However, some studies have reported that BZR1 promotes the accumulation of carotenoids in tomato fruits by directly activating the expression of *PSY* [23, 27], while the effects of BZR1 on biosynthetic genes such as *CRTISO* are rarely reported. In our previous study, we created Chinese kale *boacrtiso* mutants using CRISPR/Cas9-mediated gene editing technology, and found that the total carotenoid content of the mutants was about 11% lower than that of wild mustard, and even the total carotenoid content of M6 mutant was 25% lower than that of wild mustard, which proved the important role of *BoaCRTISO* in the process of carotenoid biosynthesis of Chinese kale [3]. It seems that the promotion of carotenoid biosynthesis medited by BoaBZR1.1 targeting *BoaCRTISO* is a key pathway that is different from that in tomato fruits. Interestingly, the overexpression of *BoaBZR1.1* in Chinese kale increased both the content of carotenoids and chlorophylls, while overexpression of *SlBZR1* in tomato fruits promoted the biosynthesis of carotenoids in tomato fruits, accompanied by the degradation of chlorophylls, thus accelerating the color transformation of tomato fruits [23, 27]. These differences suggested the mechanism that BZR1 regulates biosynthesis of photosynthetic pigments in vegetative and storage organs were different and need further study.

BoaBZR1.1 can mediate BR to regulate carotenoid biosynthesis in Chinese kale, but the response of carotenoid biosynthesis genes to BR and BoaBZR1.1 was different. In this study, the expression of *BoaCRTISO* and *BoaPSY* in Chinese kale was upregulated after the overexpression of *BoaBZR1.1*, and the content of β-carotene increased, which was consistent with the research results in citrus [39], tomato [23, 27], and pepper fruits [7] overexpressed by *BZR1*. Exogenous application of EBR elevated the expression of *BoaCRTISO* and *BoaPSY*; however, it was noteworthy that the β-carotene level was not changed. Further analysis showed that overexpression of *BoaBZR1.1* up-regulated the expression of *BoaPSY*, *BoaCRTISO*, and *BoaLCYb*, and promoted the biosynthesis of β-carotene. However, due to the expression of *BoaZEP* not changing significantly, the decomposition of β-carotene did not accelerate, which eventually led to the increase of β-carotene content. EBR treatment promoted the expression of *BoaPSY*, *BoaCRTISO*, and *BoaLCYb*, which was beneficial to the accumulation of β-carotene, and the expression of *BoaZEP* also increased at the same time, and finally led to no significant change in β-carotene content. Therefore, we think that the response of genes in carotenoid metabolic pathway to BR and BZR1.1 is not completely consistent.

Members of the same gene family often serve distinct functions and display unique expression patterns [40]. For example, in persimmon, *DkBZR1* and *DkBZR2* were expressed in various tissues. *DkBZR1* predominated in fruit tissue, while *DkBZR2* dominated in the calyx. These genes interacted antagonistically to regulate fruit softening [41]. Moreover, the copy number of a gene can vary between species, resulting in differences in expression and function among copies. In Chinese kale, three *BoaAOP2* copies were discovered. *BoaAOP2.1* and *BoaAOP2.3* exhibit functional redundancy in aliphatic glucosinolate (GSL) biosynthesis, with *BoaAOP2.1* being the most effective. Notably, *BoaAOP2.2* does not participate in the aliphatic GSL pathway [42]. In *A. thaliana*, there is only one copy of *PSY*. However, we cloned three *BoaPSYs* genes in Chinese kale, named *BoaPSY1*, *BoaPSY2*, and *BoaPSY3*. Among these three genes, only *BoaPSY2* can be directly activated by BoaBZR1.1, suggesting that the regulatory mechanisms of these three genes on carotenoid biosynthesis in Chinese kale are different (Fig. 3). Unlike *A. thaliana* and tomato, which each possess a single copy of *BZR1*, Chinese kale harbors three distinct *BoaBZR1* genes. *BoaBZR1.2* and *BoaBZR1.3* share high homology and exhibit nearly identical expression patterns. However, *BoaBZR1.1* differs in domain structure and expression pattern. Furthermore, *BoaBZR1.1* displayed a more pronounced response to EBR and within the first 6 hours, exogenous EBR and Brz treatments, respectively, induced and suppressed *BoaBZR1.1* expression. *BoaBZR1.2* responded to EBR treatment only after 3 hours. Additionally, the expression of *BoaBZR1.3* was solely enhanced by EBR, with no inhibitory effect from Brz treatment. Subsequent analysis confirmed that only BoaBZR1.1 could directly activate the transcription of *BoaCRTISO* and *BoaPSY2* (Fig. ure S4, see online supplementary material)*.* Collectively, these results indicated that *BoaBZR1.1* likely plays a distinct role in plant growth and development, especially in the context of BR-induced carotenoid biosynthesis, setting it apart from BoaBZR1.2 and BoaBZR1.3.

**Figure 6:**
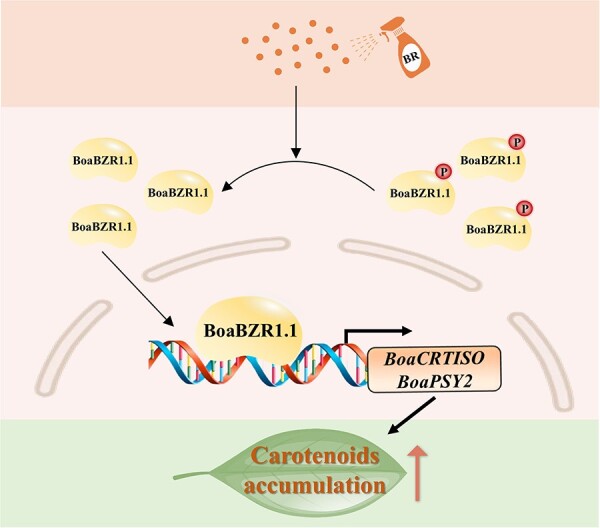
Proposed model explaining how the BoaBZR1.1 mediates BR to regulate carotenoid biosynthesis in Chinese kale.

In summary, we reported the regulatory mechanism of BR on carotenoid biosynthesis via BoaBZR1.1 in Chinese kale (Fig. 6). Initially, exogenous BR treatment triggers the activation of BoaBZR1.1, facilitating its direct interaction with the promoters of *BoaCRTISO* and *BoaPSY2*, subsequently leading to the upregulation of *BoaCRTISO* and *BoaPSY2* expression. As a result, carotenoid biosynthesis was stimulated, ultimately augmenting carotenoid levels in Chinese kale. Our findings offer fresh insights into how BR promotes carotenoid biosynthesis in *Brassica* plants and provide a foundational basis for further investigations into carotenoid biosynthesis in the vegetative organs of leafy green vegetables.

## Material and methods

### Plant materials and the conditions for cultivation

The Chinese kale cultivar ‘Sijicutiao’ underwent cultivation in growth chambers with a 12-hour light/12-hour dark photoperiod at 23 ± 1°C, accompanied by a light intensity of 36 μmol m^−2^ s^−1^. Mature leaves were harvested for the cloning of *BoaBZR1* genes. RNA extraction occurred across various Chinese kale organs during distinct developmental stages, including germinating seeds, cotyledons, true leaves, mature leaves, petioles, roots, bolting stems, inflorescences, flower buds, and fruit pods, followed by subsequent qPCR analysis.

### Exogenous BR treatment

We selected plump seeds and sowed them in 32-hole plugs, which were then placed in growth chambers. Once the Chinese kale had grown three leaves and one shoot, we added 1000 mL of water, along with 1 μM of EBR and 1 μM of Brz, to the trays at the bottom of the plugs. We harvested the second euphyllas (counting from the bottom to the top) at time points of 0 h, 3 h, 6 h, 12 h, and 24 h to assess the expression of genes related to carotenoid and chlorophyll. Additionally, we collected samples at 2 days to measure carotenoid and chlorophyll contents.

### Determination of carotenoids and chlorophylls

The composition and contents of carotenoid and chlorophyll were assessed following the procedures outlined by Sun *et al.* [3]. Chinese kale leaves were extracted using acetone. Subsequently, the samples underwent sonication, centrifugation, filtration, and were analysed via high-performance liquid chromatography (HPLC). Following separation and elution, absorbance readings were taken at 448 and 428 nm.

### Gene cloning and sequence analysis

Using the *Brassica* database (BRAD) (http://brassicadb.cn) for sequence information on *CRTISO* promoters from related species such as cabbage and Chinese cabbage, specific primers were crafted for the *BoaCRTISO* promoter. The specific primers for *BoaBZR1* genes were designed using the same approach (see Table S1, see online supplementary material). Subsequently, the *BoaCRTISO* promoter and three *BoaBZR1* genes, namely *BoaBZR1.1*, *BoaBZR1.2*, and *BoaBZR1.3*, were cloned from Chinese kale. PCR amplification was conducted and subjected to Sanger sequencing, which was performed by Tsingke Biotechnology (Beijing) Co., Ltd. To anticipate *cis*-acting elements within the *BoaCRTISO* promoter sequences, the PlantCARE online tool (http://bioinformatics.psb.ugent.be/webtools/plantcare/html/) was employed. The amino acid sequences of the three genes were compared, and the domains of BoaBZR1 were forecasted using online tools such as https://www.ncbi.nlm.nih.gov/Structure/cdd/wrpsb.cgi and https://www.bioinformatics.nl/cgi-bin/emboss/epestfind. Protein sequences of BZR1s were retrieved from BRAD for *Brassica rapa*, *B. juncea*, and *B. oleracea*, as well as from the *Arabidopsis* Information Resource (TAIR) (https://www.arabidopsis.org/) for *Arabidopsis*. Subsequently, MEGA 11.0 was employed to align the sequences and construct a phylogenetic tree using the neighbor-joining (NJ) method.

### Subcellular localization of BoaBZR1 proteins

The coding sequences of BoaBZR1s, devoid of stop codons, were isolated and incorporated into the GFP fusion vector, resulting in the construction of pC2300-35S-BoaBZR1s-eGFP. To ascertain the subcellular localization of BoaBZR1s, GFP expression was transiently observed in Chinese kale mesophyll protoplast cells, following the methodology outlined in our prior study [3].

### Yeast one-hybrid (Y1H) assay

The promoter sequence of *BoaCRTISO* in Chinese kale, spanning 1789 bp, was divided into three segments. Subsequently, pAbAi yeast bait vectors were constructed for each of these segments. The yeast one-hybrid library screening assay utilized the Matchmaker® Gold Yeast One-Hybrid Library Screening System from TaKaRa, Japan.

To confirm the interaction between BoaBZR1s and *BoaCRTISO*, the coding sequences (CDSs) of the *BoaBZR1s* genes were inserted into the pGADT7 vector. Additionally, the first fragment of the *BoaCRTISO* promoter was inserted into the pAbAi vector. In accordance with the Matchmaker® Gold Yeast One-Hybrid Library Screening System instructions from TaKaRa, Japan, the recombinant plasmids were used to transform Y1H Gold yeast cells. Subsequently, screening of the transformed yeast cells was conducted on SD/Leu-media supplemented with 250 ng ml^−1^ aureobasidin A.

### Dual-luciferase reporter (LUC) assay

The dual-luciferase assay was utilized to validate the binding of BoaBZR1s to *BoaCRTISO* promoter. To create the reporter construct, the first fragment of *BoaCRTISO* promoter was inserted into the pGreen II0800-LUC vector, and the CDSs of *BoaBZR1s* was inserted into the pGreen II002962-SK vector to form the effector constructs and subsequently transformed them into *Agrobacterium* train GV3101, and a mixture of *A. tumefaciens* carrying the reporter or effector constructs was infiltrated into *Nicotiana benthamiana* leaves. *N. benthamiana* plants that were infiltrated were subjected to 24 hours of dark treatment, followed by 24 hours of light exposure. The activity of the promoter was quantified by calculating the ratio of firefly luciferase (LUC) enzyme activity to the internal reference renilla luciferase (REN) using a multifunctional microplate reader (Thermo Scientific™, Waltham, Massachusetts, USA). The LUC/REN value in the absence of BoaBZR1s was established as one. Luciferase activities were measured using the GelView 6000Plus Intelligent image workstation (BLT, China).

### Genetic transformation of *BoaBZR1.1*

The CDS of *BoaBZR1.1* was amplified using primers containing *BamH*I and *Kpn*I restriction sites. After confirming the fragment, it was integrated into the pCAMBIA1301-35S-Nos vector at the *BamH*I and *Kpn*I sites, leading to the generation of the pCAMBIA1301-*BoaBZR1.1* construct. For the transformation of Chinese kale plants, *A. tumefaciens*-mediated techniques were applied. The pCAMBIA1301-*BoaBZR1.1* construct or an empty vector was introduced into GV3101 through a freeze–thaw method. Subsequently, the *A. tumefaciens* liquid was propagated with a dilution factor of 1:100.

For the transformation of Chinese kale calli, 1-month-old calli were co-cultured with *A. tumefaciens* carrying either the pCAMBIA1301-*BoaBZR1.1* construct or the empty vector. The calli were co-cultured on MS medium supplemented with 0.5 mg L^−1^ 1-Naphthaleneacetic acid and 2 mg L^−1^ 6-butyric acid for a duration of 2 days at room temperature. Subsequently, the calli were rinsed three times with sterile water and transferred to selective media supplemented with 200 mg L^−1^ carbenicillin and 300 mg L^−1^ timentin for transgene selection. Following this, the transgenic calli were further cultivated in selective media with the corresponding antibiotic concentrations.

The Agrobacterium-mediated transformation of Chinese kale followed the procedure outlined by Sun *et al.* [3]. Sterile Chinese kale seedlings were cultured and the cotyledons were used as explants. After infection with *A. tumefaciens* carrying either the pCAMBIA1301-*BoaBZR1.1* construct or empty vector, the cotyledons were co-cultured with *A. tumefaciens* for 3 d in MS media consisting of 0.03 mg L^−1^ naphthaleneacetic acid (NAA), 0.75 mg L^−1^ boric acid (BA), and 0.8% Phytagar. Then the explants were transferred to co-culture medium supplemented with 325 mg L^−1^ carbenicillin, and 325 mg L^−1^ timentin for 7 d. Hygromycin-resistant shoots were selected using 12 mg L^−1^ hygromycin B and were transferred to tissue culture bottles that contained subculture media. After 3 months, hygromycin-resistant plantlets were obtained and were transplanted into trays containing a mixture of peat and vermiculite (3:1) and cultured for an additional six weeks for subsequent analysis.

### Color measurements

The colors of the wild-type (WT), empty vector (EV), and the T1 generation of *BoaBZR1.1* overexpressed Chinese kale (*BoaBZR1.1*-OE) were assessed when they reached the stage of having five true leaves. Randomly, four locations on the third true leaf of each plant were chosen, and the color values in terms of L*, a*, and b* were recorded.

### RNA extraction and qPCR analysis

Total RNA was extracted and converted into cDNA. The qPCR primers for carotenoid and chlorophyll biosynthetic genes in Chinese kale were designed based on *B. oleracea* primer sequences obtained from the qPCR primer database (https://biodb.swu.edu.cn/qprimerdb/), with the exceptions of the *CLH2* and *NYC* genes [43] (Table S2, see online supplementary material). *β-actin* [44] served as the internal reference gene in the qPCR reaction. Subsequently, the relative expression level of carotenoid and chlorophyll biosynthetic genes were determined utilizing the 2^–ΔΔCT^ method.

### Statistical analysis

The results, expressed as means ± SD from three replicates, underwent statistical analysis using Excel 2021 and DPS 9.01 software. Significance among experimental sets was determined through one-way analysis of variance, followed by the least significant difference test at a 0.05 significance level.

## Supplementary Material

Web_Material_uhae104
