# Supplementary material for: BoaBZR1.1 mediates brassinosteroid-induced carotenoid biosynthesis in Chinese kale
Source: Hortic Res. 2024 Apr 9;11(6):uhae104. doi: 10.1093/hr/uhae104 (PMC11179724; doi:10.1093/hr/uhae104)
Supplement: Web_Material_uhae104 [file web_material_uhae104.zip › zhang et al. supplementary data.docx]

**Supplementary Table S1: Primers used for cloning, vector construction and mutation detection.**

| Primer names | Sequence of primers (5’-3’) | Aims |
| --- | --- | --- |
| *BoaCRTISO pro*-clone-F | GCCCATACCTACTCCTAGTAGTACTACGA | Molecular cloning of  *BoaCRTISO* promoter |
| *BoaCRTISO pro*-clone-R | AGCTGCGATCAGCACACGTTA |  |
| *BoaBZR1.1*-clone-F | ATGGTACATCGTCTAGAGACACTCCT | Molecular cloning of  *BoaBZR1s* genes |
| *BoaBZR1.1*-clone-R | TCAACCACGAGCCTTCTTCCCGTTA |  |
| *BoaBZR1.2*-clone-F | ATGACGTCGGATGGGGCTACAACAT |  |
| *BoaBZR1.2*-clone-R | TCAACCACGAGCCTTGCCGTTTCCAAGA |  |
| *BoaBZR1.3*-clone-F | ATGACGTCAGATGGAGCTACGTCGC |  |
| *BoaBZR1.3*-clone-R | TCAACCACGAGCCTTGCCGTTTCCAAGT |  |
| pABAi-*BoaCRTISO pro1*-F | cttgaattcgagctcggtaccTAATGTTAATTTTTTTTTGTCATCTGATAA | Construction of Y1H Vector of *BoaBZR1s* gene |
| pABAi-*BoaCRTISO pro1*-F | agcacatgcctcgaggtcgacGGCTCTGAGCTTGGTTCGTCG |  |
| pGADT7-*BoaBZR1.1*-F | gccatggaggccagtgaattcATGGTACATCGTCTAGAGACACTCCTT |  |
| pGADT7-*BoaBZR1.1*-R | cagctcgagctcgatggatccTCAACCACGAGCCTTCTTCCC |  |
| pGADT7-*BoaBZR1.2*-F | gccatggaggccagtgaattcATGACGTCGGATGGGGCT |  |
| pGADT7-*BoaBZR1.2*-R | cagctcgagctcgatggatccCTCAACCCACGAGCCTTGC |  |
| pGADT7-*BoaBZR1.3*-F | gccatggaggccagtgaattcCATGACGTCAGATGGAGCTACG |  |
| pGADT7-*BoaBZR1.3*-R | cagctcgagctcgatggatccTCTCCTTCCCACGGCTTAACT |  |
| pGADT7-F | TAATACGACTCACTATAGGG |  |
| pGADT7-R | TGAACTTGCGGGGTTTTTC |  |
| pABAi-F | GTTCCTTATATGTAGCTTTCGACA |  |
| pABAi-R | CCATCTCGAAAAAGGGTTTGCC |  |
| *BoaCRTISO pro1*-KpnI-F | ctatagggcgaattgggtaccCTTTATGTCCTTTTCGCGCC | Construction of LUC Vector of BoaBZR1s Gene |
| *BoaCRTISO pro1*-HindⅢ-R | caggaattcgatatcaagcttGGCTCTGAGCTTGGTTCGTCG |  |
| *BoaBZR1.1*-HindⅢ-F | caggaattcgatatcaagcttATGGTACATCGTCTAGAGACACTCCTT |  |
| *BoaBZR1.1*-KpnI-R | tgatttcagcgaattggtaccTCAACCACGAGCCTTCTTCCC |  |
| *BoaBZR1.2*-HindⅢ-F | caggaattcgatatcaagcttATGACGTCGGATGGGGCT |  |
| *BoaBZR1.2*-KpnI-R | tgatttcagcgaattggtaccTCAACCACGAGCCTTGCCG |  |
| *BoaBZR1.3*-HindⅢ-F | caggaattcgatatcaagcttATGACGTCAGATGGAGCTACGTC |  |
| *BoaBZR1.3*-KpnI-R | tgatttcagcgaattggtaccTCAACCACGAGCCTTGCCG |  |
| pGreen II002962-SK-F | TGACGCACAATCCCACTATCCTT |  |
| pGreen II002962-SK-R | TCCCTTATCGGGAAACTACTCACAC |  |
| pGreen II0800-LUC-F | GTTTTCCCAGTCACGACGTT |  |
| pGreen II0800-LUC-R | GCCTTATGCAGTTGCTCTCC |  |
| *BoaBZR1.1*-qPCR-F | GGCTACAATACCTGAGTGTGAT | Quantitative analysis of *BoaBZR1s* gene |
| *BoaBZR1.1*-qPCR-R | TAAACTCAGAGCCTTGACCAAA |  |
| *BoaBZR1.2*-qPCR-F | CCATCGCAGCAAAGATATACAC |  |
| *BoaBZR1.2*-qPCR-R | GTAAGAAGAGATGATGGGACTTTGA |  |
| *BoaBZR1.3*-qPCR-F | CTTCCTCTCTTCCTTCCCTCAG |  |
| *BoaBZR1.3*-qPCR-R | AAAGGATAGTTAAAGGACGCCA |  |
| *BoaBZR1.1*-BamHI-F | ggacagggtacccggggatccATGGTACATCGTCTAGAGACACTCCTT | Construction of subcellular localization vector of *BoaBZR1s* gene |
| *BoaBZR1.1*-SalI-R | caccatggtactagtgtcgacACCACGAGCCTTCTTCCCG |  |
| *BoaBZR1.2*-BamHI-F | ggacagggtacccggggatccATGACGTCGGATGGGGCT |  |
| *BoaBZR1.2*-SalI-R | caccatggtactagtgtcgacACCACGAGCCTTGCCGTT |  |
| *BoaBZR1.3*-BamHI-F | ggacagggtacccggggatccATGACGTCAGATGGAGCTACGTC |  |
| *BoaBZR1.3*-SalI-R | caccatggtactagtgtcgacACCACGAGCCTTGCCGTT |  |
| PC2300-F | ttcatttcatttggagaggacag | Subcellular localization vector primer |
| PC2300-R | gtgcagatgaacttcagggtcag |  |
| *BoaBZR1.1*-KpnI-F | CTCGAGGGGGGGCCCGGTACCATGGTAC | Construction of overexpression vector of *BoaBZR1s* gene |
|  | ATCGTCTAGAGACACTCCTT |  |
| *BoaBZR1.1*-BamhI-R | GATCTGCAGCCCGGGGGATCCTCAACCA |  |
|  | CGAGCCTTCTTCCC |  |
| *BoaBZR1.2*-KpnI-F | CTCGAGGGGGGGCCCGGTACCATGACGT |  |
|  | CGGATGGGGCT |  |
| *BoaBZR1.2*-BamhI-R | GATCTGCAGCCCGGGGGATCCTCAACCAC |  |
|  | GAGCCTTGCCG |  |
| *BoaBZR1.3*-KpnI-F | CTCGAGGGGGGGCCCGGTACCATGACGTC |  |
|  | AGATGGAGCTACGTC |  |
| *BoaBZR1.3*-BamhI-R | GATCTGCAGCCCGGGGGATCCTCAACCAC |  |
|  | GAGCCTTGCCG |  |
| PM1301-F | CGGGGGACTCTAGTTTATCAAG | Overexpression vector primer |
| PM1301-R | TTTGAACGATCTGCAGCCC |  |
| HYG-F | CGATTGCGTCGCATCGACC | Hygromycin detection primer |
| HYG-R | TTCTACAACCGGTCGCGGAG |  |
| GUS-F | GTCGCGCAAGACTGTAACCA | GUS detection primer |
| GUS-R | GCTAACGTATCCACGCCGTA |  |

**Supplementary Table S2: Primers used in this study for quantitative analysis.**

| Genes | Forward primer | Reverse prime |
| --- | --- | --- |
| *β-actin* | CCAGAGGTCTTGTTCCAGCCATC | GTTCCACCACTGAGCACAATGTTAC |
| *PSY1* | AAGGGCTGTAGAGTCTTCTAGA | CGTTTTTGTTGTTTGCTTCCTC |
| *PSY2* | GGGACCTTGATGATCTGAAGAA | TTCACCCAACAAACTCAAACTC |
| *PDS1* | GATCTCTTCACAAGCGCTTAAG | GCTTCCAAGAAATTGACAGTGT |
| *ZISO* | CGTTGTTTGGATCGATAACTCC | GCTAATCCACTATGCACAGTTG |
| *CRTISO* | ATATCCACACAAACTACCTGCA | AGATTGGGAGGGACTCACTCCA |
| *LCYe2* | CAGGTTCCGGTATAGAGAGTTG | ACGTATAGAATCTCCGAACCAC |
| *LCYb* | GTTGTTGATCTAGCTATCGTTGGC | GAGTTTGGGGGAAGGATCGAT |
| *ZEP2* | AGAACTCCTAGTTTCACTTGGG | TATCCTCAATGGCCATACATCC |
| *VDE* | AAGGCATTTCCTCACATCCTTA | ACCAAGAAAGTGCCTTTGATTC |
| *NXS* | CAGTTGACATGCCAGCAAGTCC | CGAATCGGATGATACACTGGGGA |
| *ALAD* | AGCTTCCCATCGATCAAAGTAA | GAAGTTGCAACTGGAAGAGAAG |
| *HemE* | ATGTTACTCCTCAGGCTTATCG | AGAGTTAGTGTGTCTTCTTGGG |
| *ChlH* | CACTACCACCAAACACTCATTC | GAGACAGAGGACTTCACTTTGA |
| *CS* | GGCGGAGACTGATACAGATAAA | TACCAAGAAGCTGGTTAATGCT |
| *PaO* | TCTCGATTTCTCCTCAAAGACC | TGAACTCAGACCCTTCTTCTTC |
| *PPH* | AGAGGTTATCGGTGAGCCA | GACGAGATGAGGATGGG |
| *NYC* | TTACATCTCGCAGTTCTGA | GCAATACCAACTACCTTAGC |


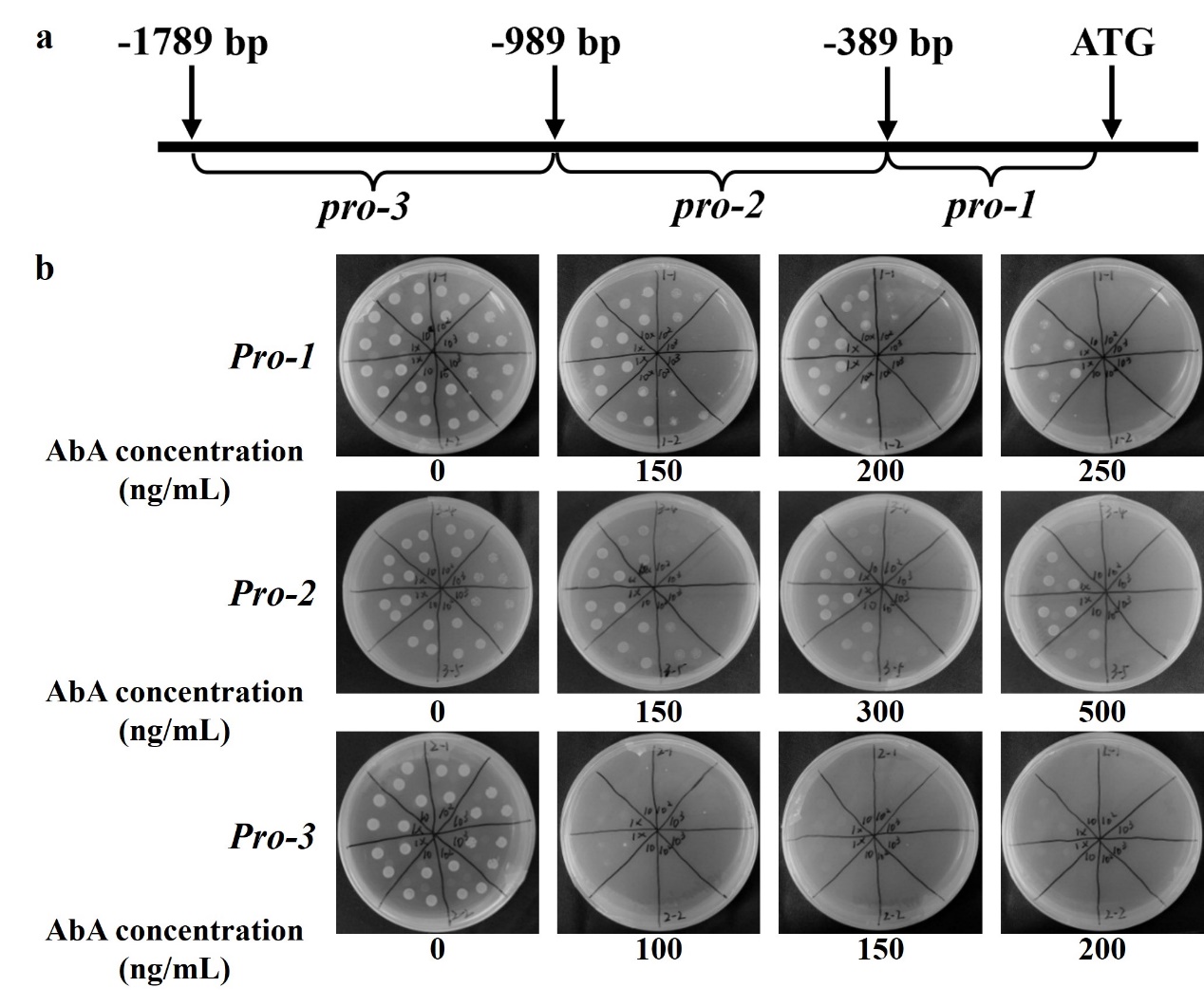


**Supplementary Fig. S1** **Truncation and autoactivation test of *BoaCRTISO* promoter.**

**a** The promoter sequence of *BoaCRTISO* (1789 bp) in Chinese kale was divided into three segments (389 bp, 600 bp and 800 bp). **b** Determining the minimal inhibitory concentration of Aureobasidin A for the three fragments of *BoaCRTISO* promoter.


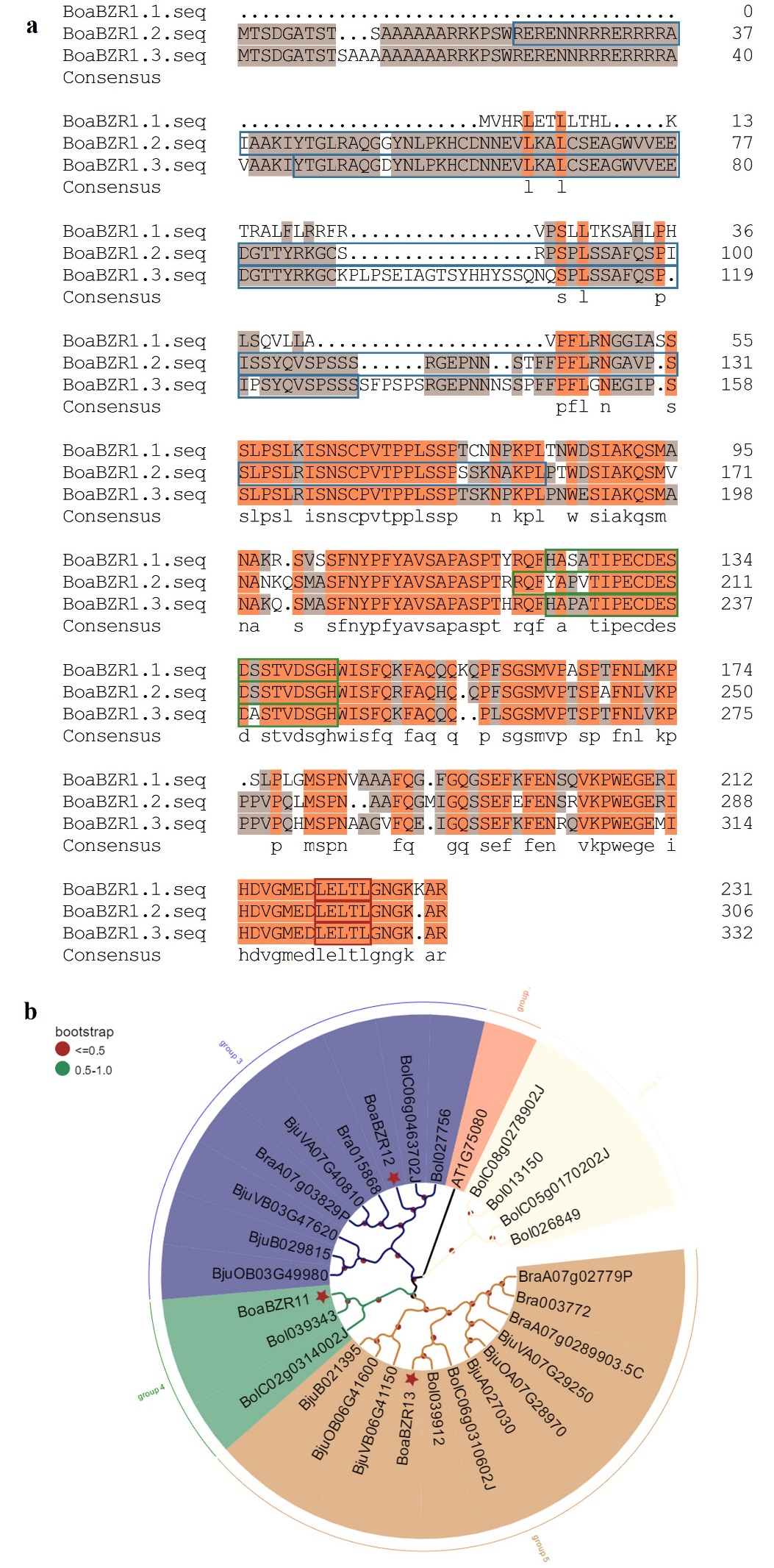


**Supplementary Fig. S2** **Characterization of the BoaBZR1s.**

**a** Amino acid sequence alignment of BoaBZR1s. The area marked by the blue box represented the BES1_N super family domain; The area marked by the green box represented the PEST domain; The area marked by the red box represented the EAR domain. **b** Phylogenetic tree of BoaBZR1s.


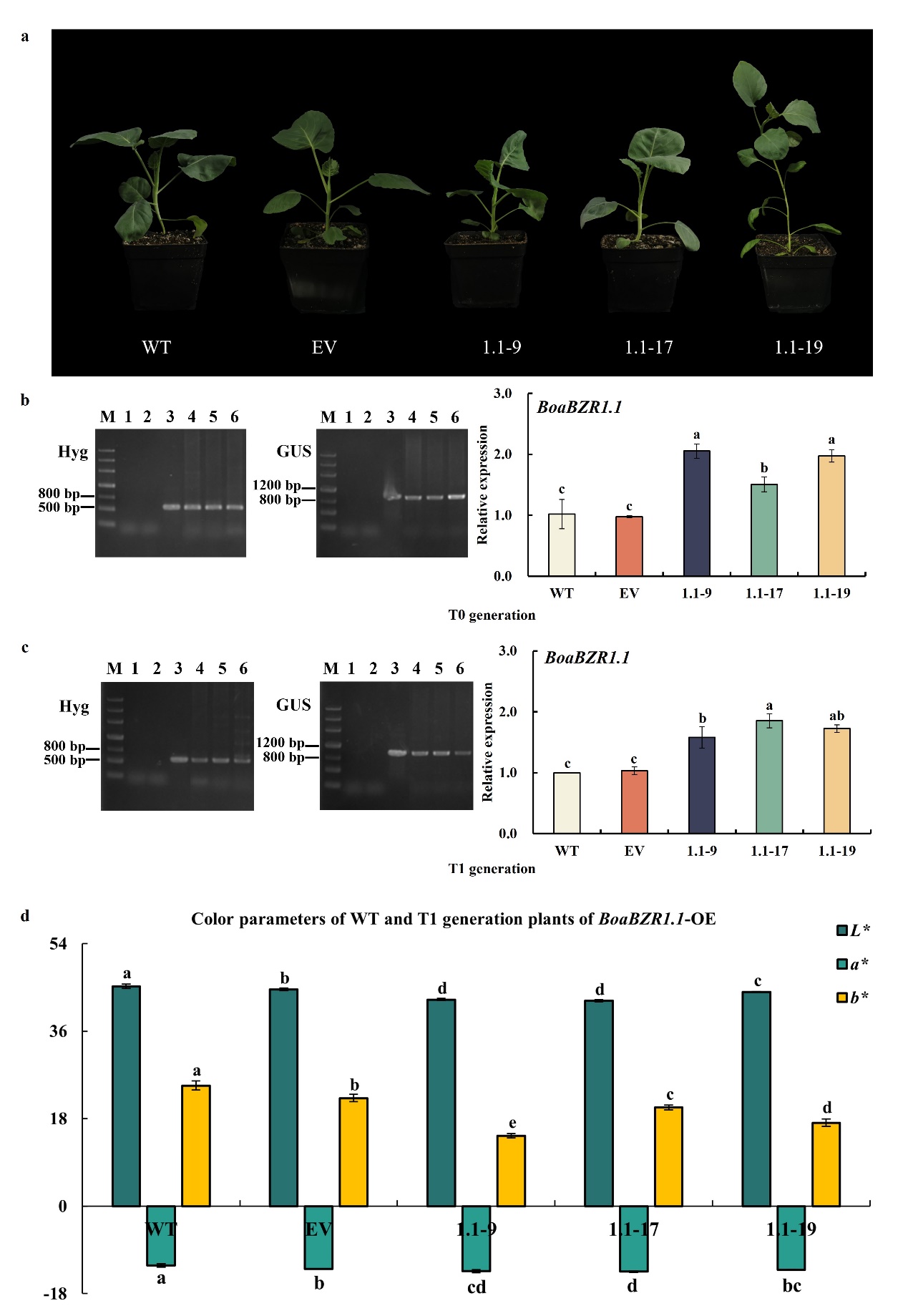


**Supplementary Fig. S3** ***BoaBZR1.1* promoted the biosynthesis of carotenoids and chlorophyll in Chinese kale.**

**a** Phenotypes of T0 generation of *BoaBZR1.1*-OE Chinese kale. **b** Tag detection of resistant plants and the expression level of *BoaBZR1.1* in T0 generation. **c** Tag detection of resistant plants and the expression level of *BoaBZR1.1* in T1 generation. **d** Color parameters of WT and T1 generation plants of *BoaBZR1.1-OE*. The data were expressed as the means ± SDs. The same letter in the same histogram indicated that there was no significant difference between the values tested, according to the LSD (*p* < 0.05).


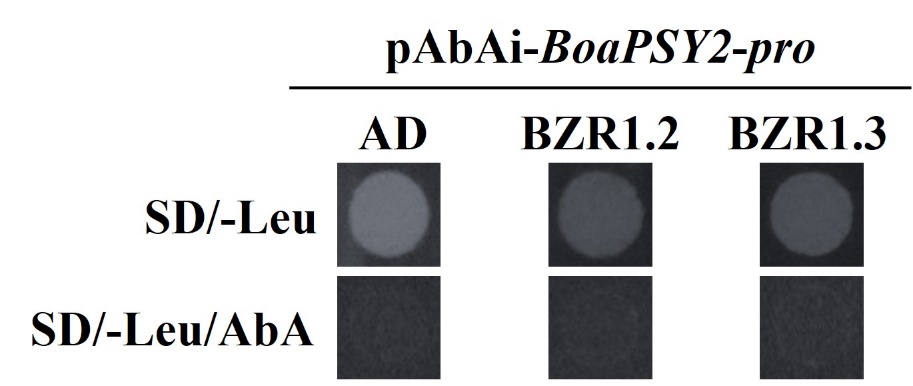


**Supplementary Fig. S4** **BoaBZR1.2 and BoaBZR1.3 could not directly combined with the promoter of *BoaPSY2*.**
